# GRIEF COACH, A TEXT-BASED GRIEF SUPPORT INTERVENTION: ACCEPTABILITY AMONG HOSPICE FAMILY MEMBERS

**DOI:** 10.1093/geroni/igae098.0958

**Published:** 2024-12-31

**Authors:** Emma Payne, Melissa Lunardini

**Affiliations:** Help Texts, Seattle, Washington, United States; Help Texts, San Diego, California, United States

## Abstract

Although older adults experience more bereavement, they are less likely to seek support. This poses significant risks, including increased risk for prolonged grief disorder, mood disorders, loneliness, and other adverse health outcomes (Ghesquiere, 2013). Limited service access and uptake among older populations can be attributed to factors such as inadequate Medicare coverage, shortage of trained providers, and a dearth of bereavement care options tailored to the unique preferences and needs of older adults (Bartels, 2003). Developing age-appropriate, accessible grief interventions is key to addressing the service gap and associated vulnerabilities related to grief. This presentation will report on grievers’ perceptions of the acceptability and helpfulness of Grief Coach, an innovative text-based grief support service aligned with a public health approach to bereavement care. The text messages are grounded in contemporary models of bereavement and coping, provide supportive education, and encourage engagement in adaptive coping behaviors. Participants in the evaluation were bereaved family members who received Grief Coach as a hospice benefit. The 13-month program retention rate was 86%. Among subscribers who met inclusion criteria for and completed a brief evaluation (N=100, response rate = 65%), 73% rated the program as “Very helpful” and 74% rated it as contributing “A great deal” or “Considerably” to their sense of being supported in their grief. Older grievers (age 65+ years old) and males tended to give the highest ratings. These preliminary data suggest that the Grief Coach text message program may be an acceptable and helpful way to support the aging grieving population.

